# Prevalence of high blood pressure subtypes and its associations with BMI in Chinese children: a national cross-sectional survey

**DOI:** 10.1186/s12889-017-4522-2

**Published:** 2017-06-26

**Authors:** Yide Yang, Bin Dong, Shuo Wang, Yanhui Dong, Zhiyong Zou, Lianguo Fu, Jun Ma

**Affiliations:** 1 Institute of Child and Adolescent Health, School of Public Health, Peking University Health Science Center, 38 Xue Yuan Road, Haidian District, Beijing, 100191 China; 2 Centre for Chronic Disease, School of Medicine, The University of Queensland, Brisbane, Australia; 3 Department of Preventive Medicine, Bengbu Medical College, Bengbu, Anhui China

**Keywords:** Blood pressure, Children, High blood pressure subtype, Obesity, Body mass index

## Abstract

**Background:**

Data on prevalence and characteristics of different high blood pressure subtypes are lacking among Chinese children. Regarding the mechanistic differences between isolated systolic high blood pressure and isolated diastolic high blood pressure and their different impact on end organ diseases, it is necessary to examine the prevalence of different high blood pressure subtypes in Chinese children and explore their associations with adiposity.

**Methods:**

Data were derived from the baseline data of a multi-centered cluster randomized controlled trial involving participants from China. High blood pressure was defined according to age-, gender- and height-specific 95th percentile developed by the National High Blood Pressure Education Program Working Group. Body mass index was used to classify underweight, normal weight, overweight and obesity.

**Results:**

The prevalence of HBP was 10.2% and 8.9% for boys and girls, respectively. Isolated systolic high blood pressure is the dominant high blood pressure subtype among Chinese boys aged 6–17 years and girls aged 12–17 years, while isolated diastolic high blood pressure was the most common high blood pressure subtype in girls aged 6–11 years. In boys, the status of overweight doubled the risk of isolated systolic high blood pressure (95% CI, 1.73, 2.31; *P* < 0.001) compared with the normal weight group, and the risk for obese children was 4.32 (95% CI, 3.81, 4.90; *P* < 0.001). The corresponding odds ratios in girls were 2.04 (95% CI, 1.68, 2.48, *P* < 0.001) for overweight, and 4.0 (95% CI, 3.36, 4.76, *P* < 0.001) for obesity. Similar patterns were also observed in the association between combined systolic and diastolic high blood pressure and adiposity.

**Conclusion:**

The distribution of high blood pressure subtypes in boys differed from those in girls, and boys with adiposity showed a higher risk of high blood pressure than their female counterpart. Difference in strength of association between isolated diastolic high blood pressure and isolated systolic high blood pressure with body mass index was also found. These results may aid current strategies for preventing and controlling pediatric hypertension.

**Electronic supplementary material:**

The online version of this article (doi:10.1186/s12889-017-4522-2) contains supplementary material, which is available to authorized users.

## Background

Elevated blood pressure (BP), especially high systolic blood pressure (SBP), is the leading cause of chronic disease worldwide. It is estimated that high SBP accounts for 10.4 million deaths and 208.1 million disability-adjusted life-years [1, 2]. Although current strategies of prevention, controlling and treatment focus on adults [3, 4], pediatric high blood pressure (HBP), which could track into adulthood [5, 6] and result in a series of end-organ damages [7], has been an alarming public health problem in China [8].

Compared with diastolic blood pressure (DBP), high SBP had a more profound effect on myocardial infarction, angina and peripheral artery disease [9]. Mechanistically, it is reported that pediatric ISHBP represented an early stage of hypertension that might be modulated by the association between obesity and sympathetic nervous system hyperactivity, while no such phenomenon was found for IDHBP [10]. In adults, isolated hypertension(ISH) increases steadily with age, which is different from IDHBP [11]. However, it is unclear whether this age-related change in HBP exists in children as well.

To date, the prevalence of different HBP subtype is unknown in Chinese children. Although the close relationship between body mass index (BMI) and HBP has been well known [12], the associations between different HBP subtypes and BMI are rarely reported. In the present study, we investigated the gender and age disparity of HBP subtype among Chinese children based on a large national survey, and examined the strength of the association between BMI and HBP subtypes. Regarding the mechanistic differences between ISHBP and IDHBP, we hypothesized that the magnitude of association between ISHBP with BMI differs from that of IDHBP. These results have a potential to improve our understanding of pediatric HBP, and aid strategies for controlling and preventing HBP in children.

## Methods

### Study design and participants

Data were derived from the baseline data of a multi-centered cluster randomized controlled trial involving Chinese children and adolescents from 7 provinces (Liaoning, Tianjin, Ningxia, Shanghai, Chongqing, Hunan and Guangdong) in September of 2013. The details of the recruitment process of participants were described in previous publication [13]. The study was approved by the Medical Ethical Committee of Peking University Health Science Center (IRB0000105213034). Written informed consent was obtained from all children and their parents. In total, 62,168 participants aged 6–17 years with complete data of body weight, height, and blood pressure were involved in the present study.

### Measurements and definitions

All participants enrolled in the current study underwent physical examination to collect anthropometric data (weight and height) according to standard procedures by trained project members [14]. Weight was measured to the nearest 0.1 kg using a lever scale with the child wearing only underwear. Height was measured to the nearest 0.1 cm using a stadiometer according to a standardized protocol. BMI (kg/m^2^) was calculated as weight (kg) divided by the height (m) squared.

Participants were defined as underweight according to the Chinese national screening criteria for malnutrition of children aged 6–18 years [15]. According to the Chinese BMI percentile criteria for screening overweight and obesity of children aged 7–18 years, the participants with an age- and gender-specific BMI ≥ 95th percentile are defined as obese, while those with an age- and gender-specific BMI between 85th and 95th percentile are defined as overweight [16], and the BMI percentile criteria of overweight(between 85th and 95th percentile) and obesity(≥ 95th percentile) for children younger than 7 years was defined according to a cut-offs criteria of a representative Chinese population [17]. After definition of underweight/overweight/obese (Additional file 1: Table S1 and Additional file 2: Table S2), other participants with an age- and gender-specific BMI less than the 85th percentiles are considered as normal-weight children.

BP was measured according to the recommendation of the National High Blood Pressure Education Program Working Group in Children and Adolescents [18] using standard clinical sphygmomanometer. BP measurements were taken 5 min after resting. If the measured difference was >10 mmHg, measurement was repeated until the final two measures differed ≤10 mmHg, and the mean of the final two measures was used in analyses. Systolic BP was defined as the onset of “tapping” Korotkoff sound (K1), and diastolic BP was defined as the fifth Korotkoff sound (K5).

High BP is defined as SBP and/or DBP ≥ the age-, gender- and height-specific 95th percentile [18]. Isolated systolic high blood pressure (ISHBP) is defined as high SBP and normal DBP. Isolated diastolic high blood pressure (IDHBP) is defined as high DBP and normal SBP. A child who was both high in systolic and diastolic high blood pressure was identified as combined systolic and diastolic high blood pressure (SDHBP).

### Statistical analysis

All data analyses were performed by SPSS for Windows (version 19.0, SPSS Inc., Chicago, IL, USA). Continuous variables were described as mean and standard deviation (s.d.) for, numerical data were described as rates. Chi square tests were used to compare the prevalence of HBP subtypes between boys and girls. Linear regression was used to analyze the associations between BMI and SBP/DBP, and β with standard error (SE) were estimated. Logistic regression was used to analyze the associations between BMI categories and HBP or different HBP subtypes, and odds ratios (ORs) with 95% CIs (confidence intervals) were also estimated.

## Results

### Characteristic of the study population

General characteristic of the population is presented in Table 1. A total of 62,168 children were involved in the present study, including 32,064 (51.6%) boys and 30,104 (48.4%) girls. As expected, boys had higher SBP and DBP than girls (*P* < 0.001). In terms of the distribution of HBP subtypes, boys had significantly higher prevalence of ISHBP than girls (4.4% and 2.9% for boys and girls, respectively) (*P* < 0.001). The prevalence of overweight and obesity were 15.0% and 13.6% in boys, and higher than those in girls (*P* < 0.001).

**Table 1:** Basic characteristics of 6–17 years old schoolchildren with different high blood pressure subtype

Variable	Total	Boys(*n* = 32,064)	Girls(*n* = 30,104)	*P*
	Mean(s.d.)	Mean(s.d.)	Mean(s.d.)	
Age	10.8(3.3)	10.7(3.3)	10.9(3.3)	<0.001
BMI	18.6(3.8)	18.8(3.9)	18.2(3.5)	<0.001
SBP	104.3(12.2)	105.7(12.5)	102.8(11.6)	<0.001
DBP	66.3(8.8)	66.8(9.0)	65.7(8.6)	<0.001
HBP	5933(9.5%)	3262(10.2%)	2671(8.9%)	<0.001
Normal BP^a^	56,235(90.5%)	28,802(89.8%)	27,433(91.1%)	
HBP subtype				
ISHBP^a^	2263(3.6%)	1404(4.4%)	859(2.9%)	<0.001
IDHBP^a^	1992(3.2%)	1001(3.1%)	991(3.3%)	
SDHBP^a^	1678(2.7%)	857(2.7%)	821(2.7%)	
nutritional status				
Underweight^a^	4456(7.2%)	2528(7.9%)	1928(6.4%)	<0.001
Normal weight^a^	43,121(69.4%)	20,365(63.5%)	22,756(75.6%)	
Overweight^a^	7888(12.7%)	4823(15.0%)	3065(10.2%)	
Obesity^a^	6703(10.8%)	4348(13.6%)	2355(7.8%)	

^a^values are numbers and proportions (%). *s.d*. standard deviation, *BMI* body mass index, *SBP* systolic blood pressure, *DBP* diastolic blood pressure, *HBP* high blood pressure, *SDHBP* combined systolic and diastolic high blood pressure, *IDHBP* isolated diastolic high blood pressure, *ISHBP* isolated systolic high blood pressure

### Prevalence of HBP subtypes and the trend with age

**Fig. 1:**
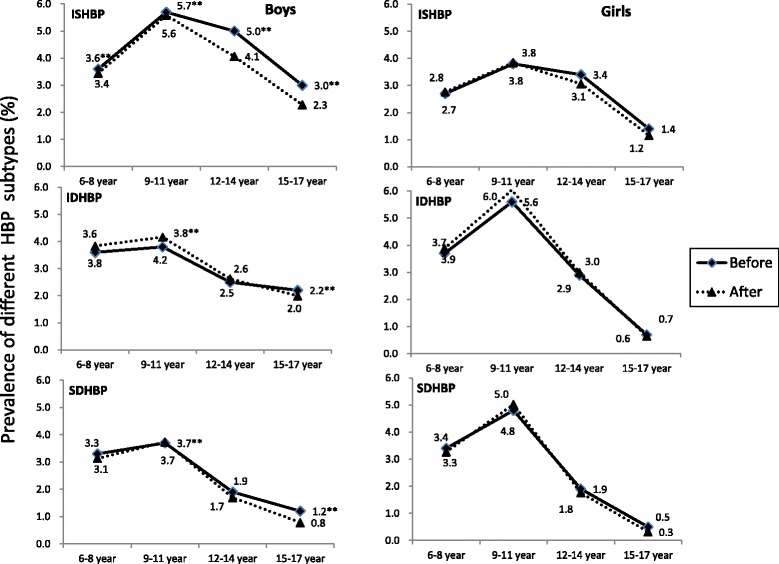
Prevalence of different high blood pressure subtype before and after adjusted for BMI and age. Showed adjusted prevalence of different HBP subtypes (%) according to age groups in Chinese 6–17 years old children, before and after adjusted for BMI and age. ISHBP (isolated systolic high blood pressure), SDHBP (combined systolic/diastolic high blood pressure), IDHBP (isolated diastolic high blood pressure). **The original prevalence of HBP subtype is significantly different between boys and girls (≤0.001)

In total, 9.5% of the children had HBP, which were 10.2% and 8.9% for boys and girls, respectively (Table 1). In both sexes, there was an inverted U-shaped pattern in the prevalence of HBP, as well as different HBP subtypes, with age (Fig. 1, Additional file 3: Figure S1 and Additional file 4: Table S3). In general, HBP increased with age to the highest of 13.3% at 10 years old, then slowly decreased with age in boys. While in girls, HBP reached the peak prevalence (15.9%) at 10 years old, then sharply decreased with age (Additional file 4: Table S3). Boys had higher prevalence of ISHBP than girls across age groups (*P* < 0.001, Additional file 4: Table S3). In boys, HBP and different HBP subtypes (ISHBP, IDHBP and SDHBP) reached the highest prevalence (13.2%, 5.7%, 3.8% and 3.7%) among 9–11 year age group. The same pattern was found in girls, with the highest prevalence of 14.3%, 3.8%, 5.6% and 4.8% for HBP, ISHBP, IDHBP and SDHBP observed in 9–11 year age group, respectively (Fig. 1 and Additional file 3: Figure S1). After adjusting for BMI and age, the trend in the prevalence of HBP and HBP subtypes over the age groups minimally changed in both sexes (Fig. 1 and Additional file 3: Figure S1).

### Frequency distribution of HBP subtypes by age group

**Fig. 2:**
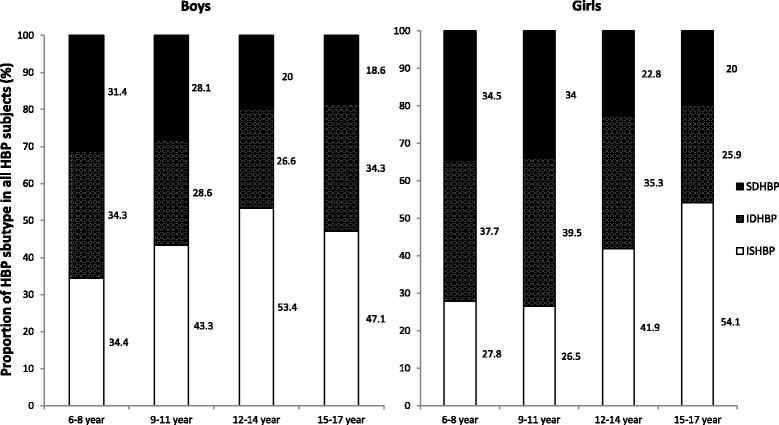
Frequency distribution of high blood pressure subtypes in Chinese children. Showed proportion of HBP subtypes (%) in Chinese children aged 6 to 17 years old with high blood pressure (HBP). SDHBP (combined systolic and diastolic high blood pressure), IDHBP (isolated diastolic high blood pressure), ISHBP (isolated systolic high blood pressure)

The most common type of HBP subtype is ISHBP for boys in all age groups, ranging between 34.4% and 53.4% (Fig. 2). Nevertheless, IDHBP was the most common HBP subtype in girls 6 to 11 years old, while ISHBP was the primary HBP subtype in 12–17 years age group (Fig. 2).

### Association between adiposity and BP/HBP/HBP subtypes

When adjusted for age, SBP is significantly associated with BMI in boys and girls (β = 1.09 and 1.01, *P* < 0.001) and similar pattern was also observed in DBP (β = 0.60 for boys and 0.57 for girls, *P* < 0.001).

For boys, BMI was significantly associated with HBP (OR = 1.15, 95% CI: 1.14, 1.16; *P* < 0.001). Consistently, in girls BMI was significantly associated with HBP (OR = 1.14, 95% CI: 1.13, 1.15; *P* < 0.001). Also, BMI was significantly associated with all HBP subtypes in boys and girls with age adjusted (all *P* < 0.001, Table 2), especially for ISHBP and SDHBP. For boys, one-unit increment of BMI was associated with 18% higher risk of ISHBP and SDHBP (95% CI: 1.16, 1.19; *P* < 0.001). For boys, BMI was also significantly associated with HBP (OR = 1.15, 95% CI: 1.14, 1.16; *P* < 0.001). In girls, one-unit increment of BMI was associated with 17% higher risk of ISHBP (95% CI: 1.15, 1.19; *P* < 0.001) and 15% higher risk of SDHBP (95% CI: 1.13, 1.18; *P* < 0.001) (Table 3). For girls, BMI was significantly associated with HBP (OR = 1.14, 95% CI: 1.13, 1.15; *P* < 0.001).

**Table 2:** Association between BMI and different BP traits

Gender	BP traits	β/OR	SE/95% CI	*P*
Boys	SBP	1.09	0.02	<0.001
	DBP	0.60	0.01	<0.001
	ISHBP	1.18	(1.16,1.19)	<0.001
	IDHBP	1.07	(1.05,1.09)	<0.001
	SDHBP	1.18	(1.16,1.19)	<0.001
	HBP	1.15	(1.14,1.16)	<0.001
Girls	SBP	1.01	0.02	<0.001
	DBP	0.57	0.02	<0.001
	ISHBP	1.17	(1.15,1.19)	<0.001
	IDHBP	1.07	(1.05,1.09)	<0.001
	SDHBP	1.15	(1.13,1.18)	<0.001
	HBP	1.14	(1.13,1.15)	<0.001

Adjusted for age. *OR* odds ratio, *BMI* body mass index, *HBP* high blood pressure, *SBP/DBP* systolic/diastolic blood pressure, *IDHBP* isolated diastolic HBP, *SDHBP* combined systolic/diastolic HBP, *IDHBP* isolated diastolic HBP, *ISHBP* isolated systolic HBP. *β and SE* standard error were obtained by linear regression analysis between BMI and SBP/DBP. OR and 95% CI were obtained by logistic regression analysis between BMI and HBP/HBP subtypes

**Table 3:** Association between BMI categories and HBP or different HBP subtypes

Gender	HBP subtypes	Nutritional status	OR	95% CI	*P*
Boys	ISHBP	Normal weight	1(ref)		
		Underweight	0.38	(0.27, 0.55)	<0.001
		Overweight	2.00	(1.73, 2.31)	<0.001
		Obese	4.32	(3.81, 4.90)	<0.001
	IDHBP	Normal weight	1(ref)		
		Underweight	0.7	(0.52, 0.93)	0.013
		Overweight	1.28	(1.08, 1.53)	0.005
		Obese	1.64	(1.38, 1.94)	<0.001
	SDHBP	Normal weight	1(ref)		
		Underweight	0.47	(0.30, 0.73)	0.001
		Overweight	2.11	(1.76, 2.54)	<0.001
		Obese	4.34	(3.71, 5.09)	<0.001
	HBP	Normal weight	1(ref)		
		Underweight	0.52	(0.43,0.64)	<0.001
		Overweight	1.75	(1.59,1.94)	<0.001
		Obese	3.31	(3.03,3.61)	<0.001
Girls	ISHBP	Normal weight	1(ref)		
		Underweight	0.52	(0.35, 0.79)	0.002
		Overweight	2.04	(1.68, 2.48)	<0.001
		Obese	4.00	(3.36, 4.76)	<0.001
	IDHBP	Normal weight	1(ref)		
		Underweight	0.62	(0.45, 0.85)	0.003
		Overweight	1.23	(1.01, 1.51)	0.038
		Obese	1.57	(1.28, 1.93)	<0.001
	SDHBP	Normal weight	1(ref)		
		Underweight	0.46	(0.30, 0.71)	<0.001
		Overweight	1.87	(1.53, 2.29)	<0.001
		Obese	3.48	(2.90, 4.16)	<0.001
	HBP	Normal weight	1(ref)		
		Underweight	0.55	(0.44,0.68)	<0.001
		Overweight	1.66	(1.47,1.87)	<0.001
		Obese	2.84	(2.54,3.18)	<0.001

Adjusted for age. *OR* odds ratio, *HBP* high blood pressure, *SDHBP* combined systolic/diastolic high blood pressure, *IDHBP* isolated diastolic high blood pressure, *ISHBP* isolated systolic high blood pressure

After adjustment for age, using the normal weight group as reference, underweight was a protective factor for the three HBP subtype in boys and girls (Table 3, all *P* < 0.05), while overweight and obesity are both risk factors for HBP and different HBP subtypes (all *P* < 0.05). For the risk of HBP, overweight boys have an OR of 1.75 (95% CI: 1.59, 1.94; *P* < 0.001), compared with the reference group, and the risk increased to 3.31 (95% CI: 3.03, 3.61; *P* < 0.001) for the obese boys. Meanwhile, overweight girls have an OR of 1.66 (95% CI: 1.47, 1.87; *P* < 0.001), and the risk increased to 2.84 (95% CI: 2.54, 3.18; *P* < 0.001) for the obese girls.

In boys, overweight group has an OR of 2.00 (95% CI: 1.73, 2.31; *P* < 0.001) for ISHBP, compared with the reference group, and the risk increased to 4.32 (95% CI: 3.81, 4.90; *P* < 0.001) for the obese group. For IDHBP, overweight and obese group had ORs of 1.28 (95% CI: 1.08, 1.53; *P* = 0.005) and 1.64 (95% CI: 1.38, 1.94; *P* < 0.001) respectively, which were lower than those for ISHBP. For SDHBP, the corresponding values for overweight and obese group are 2.11 (95% CI, 1.76, 2.54; *P* < 0.001) and 4.34 (95% CI, 3.71, 5.09; *P* < 0.001) (Table 3). Similar pattern was also observed in girls, although the associations between HBP subtypes and BMI groups were of weaker amplitude compared with those in boys.

## Discussion

The current study demonstrated that there is a gender difference of HBP subtypes distribution, and boys had a higher risk of ISHBP than girls. In boys aged 6 to 17 years old and girls aged 12 to 17 years old, ISHBP is the most common subtype of HBP in Chinese children. In girls aged 6 to 11 years old, IDHBP is the most frequent subtype. In addition, in both sexes, 9 to 11 years old were the age related to the highest prevalence of all types of HBP. These patterns were slightly changed when BMI was adjusted for. Furthermore, compared with IDHBP, a stronger association was observed between ISHBP and BMI.

To our knowledge, the present study is the first study concerning different HBP subtypes (ISHBP, IDHBP and SDHBP) prevalence and these subtypes’ associations with BMI among Chinese young population. Our team previously reported the increasing trend in HBP from 2005 to 2010 using the Chinese National Surveys on Students’ Constitution and Health (CNSSCH) [8]. That study showed that the prevalence of systolic and diastolic HBP in 2010 were 4.9% and 3.9% in boys and 3.5% and 4.0% in girls, respectively. In the present study, the prevalence of SHBP and DHBP among 6- to 17-year-old children in 2013 is higher than those in 2010, with SHBP 7.05% and 5.58% for boys and girls respectively, and DHBP is 5.79% and 6.02% for boys and girls. Additionally, there is an inverted U-shaped pattern in the prevalence of HBP with age among various HBP subtypes and sex groups, with children aged 9–11 years old had the highest prevalence. These findings were lower than those observed in Swiss adolescents, who had the prevalence of HBP and ISHBP of 11.4% and 9.60%, respectively [19]. Xu et al. used the same standard of HBP explored the prevalence of HBP by age and gender in Chinese children. In line with our results, boys had higher prevalence of HBP than girls, and HBP generally increased by age, but the trend with age were slightly different. In Xu et al.’s study boys had a peak prevalence of HBP at age of 16 years, while girls had a peak prevalence of HBP at age of 10 years [20]. In our study, the prevalence of HBP is around 10 years old in both genders (Additional file 4: Table S3). The different age with peak HBP prevalence may due to the different ethnicities. In addition, we also specified the ages with peak prevalence among each HBP subtypes, which were generally consistent with those of HBP.

In addition to previous studies that showed boys had significantly higher prevalence of HBP than girls [8, 21, 22], our results presented boys related with an enhanced risk of ISHBP across age groups. Nevertheless, the situation of IDHBP was different, which revealed girls of 6 to 14 years old had a higher risk of IDHBP than their male counterparts.

Current study depicted the distribution of HBP subtype across age groups, which were different from those in adult population [23]. Increasing evidence has showed that the impact of ISHBP on the risk of cardiovascular diseases, including coronary heart disease, stroke, and related mortality and morbidity is stronger than IDHBP [24, 25]. Moreover, high SBP could be tougher to control compared with high DBP [26]. Regarding the phenomenon that pediatric HBP could track into adulthood [5], the age and gender characteristics of prevalence of different HBP subtypes could be of vital importance when strategies for prevention and control HBP are developed in young population, which may further benefit their subsequent adulthood cardiovascular profile.

Additionally, the current study demonstrated the associations between different HBP/HBP subtypes and BMI. Regarding the association between BMI and BP, similar results were found in other studies in Chinese children. Generally, children with overweight had a doubled higher risk of HBP, while quadrupled the risk was showed in children with obesity [27–31]. Though elevated BMI is associated with a significantly increased risk of ISHBP and SDHBP, the correlation between ISHBP and BMI is much stronger than that for IDHBP. These findings were consistent with a previous study conducted in a US population [32]. The possible mechanism for these differences in the magnitude of the association might be related with central pulsatile hemodynamics. Pierce et al. found that the adolescents with high BMI had increased left ventricular mass, high aortic wave amplitude, and systolic BP, but not diastolic BP, compared with their peers with normal weight [33]. Additionally, higher fat accumulation has been reported as an important independent predictor of aortic stiffness (aortic pulse-wave velocity), which is strongly associated with SBP [34]. Therefore, our finding suggested that intervention for reducing BMI may lead to a bigger benefit in controlling of ISHBP than IDHBP.

## Strengths and limitations

The strengths of our study include that we used a relative large and representative sample. Additionally, our sample included participants with wide age range (from 6 to 17 years), which allowed us to explore the trend of pediatric HBP subtypes with age. However, there are also limitations. Firstly, the BP measurement was collected on one occasion, and the estimated prevalence of HBP may be overestimated. In the future study, BP measurements with multiple visits or using ambulatory blood pressure monitor would be better to identify HBP. Secondly, we did not collect the information about medication and salt consumption in this survey, which were warranted in our further study. Thirdly, this is a cross-sectional study which is not able to provide causal inference for the association between BMI and BP.

## Conclusion

Our study revealed that ISHBP is the predominant subtype of pediatric HBP among Chinese 6 to 17-year-old boys and 12- to 17-year-old girls, and IDHBP was the most common subtype of HBP in girls aged 6–11 years. An inverted U-shaped trend in prevalence of HBP subtypes across age was also observed. Regarding of the strength of association between ISHBP and BMI was stronger than those for IDHBP, children with ISHBP may obtain more benefices from obesity intervention. These findings may aid the strategies for preventing and controlling of hypertension in early life.

## Additional files

Additional file 1: Table S1. Body Mass Index Reference Norm for Screening Overweight and Obesity Among Chinese children aged 7–18 years (kg/m^2^). (DOC 69 kb)

Additional file 2: Table S2. Body Mass Index Reference Norm for Screening underweight Among Chinese children aged 6–17 years (kg/m2). (DOC 87 kb)

Additional file 3: Figure S1. Showed the prevalence of HBP (%) according to age groups in Chinese 6–17 years old children, before and after adjusted for BMI and age. HBP(high blood pressure). **The original prevalence of HBP subtype is significantly different between boys and girls (≤0.001). *The original prevalence of HBP subtype is significantly different between boys and girls (≤0.05). (PPT 187 kb)

Additional file 4: Table S3. The prevalence of HBP or different HBP subtypes among 6–17 years old Chinese schoolchildren. (DOC 47 kb)

## Abbreviations

BMI: Body mass index
BP: Blood pressure
DBP: Diastolic blood pressure
HBP: High blood pressure
IDH: Isolated diastolic hypertension
IDHBP: Isolated diastolic high blood pressure
ISH: Isolated systolic hypertension
ISHBP: Isolated systolic high blood pressure
SBP: Systolic blood pressure
SDH: Combined systolic and diastolic hypertension
SDHBP: Combined systolic and diastolic high blood pressure
