# Supplementary material for: Prevalence of high blood pressure subtypes and its associations with BMI in Chinese children: a national cross-sectional survey
Source: BMC Public Health. 2017 Jun 26;17:598. doi: 10.1186/s12889-017-4522-2 (PMC5485696; doi:10.1186/s12889-017-4522-2)
Supplement: Supplementary file 1 — Body Mass Index Reference Norm for Screening Overweight and Obesity Among Chinese children aged 7–18 years (kg/m2). (DOC 69 kb) [file 12889_2017_4522_MOESM1_ESM.doc]

| **Table S1. Body Mass Index Reference Norm for Screening Overweight and Obesity Among Chinese children aged 7-18 years (kg/m^2^)** | | | | | |
| --- | --- | --- | --- | --- | --- |
| **Age** | **Boys** | |  | **Girls** | |
| **(years)** | **Overweight** | **Obesity** |  | **Overweight** | **Obesity** |
| 6～ | 16.8～18.3 | ≥18.4 |  | 16.7～18.3 | ≥18.4 |
| 6.5～ | 17.0～18.7 | ≥18.8 |  | 16.8～18.5 | ≥18.6 |
| 7～ | 17.4～19.1 | ≥19.2 |  | 17.2～18.8 | ≥18.9 |
| 8～ | 18.1～20.2 | ≥20.3 |  | 18.1～19.8 | ≥19.9 |
| 9～ | 18.9～21.3 | ≥21.4 |  | 19.0～20.9 | ≥21.0 |
| 10～ | 19.6～22.4 | ≥22.5 |  | 20.0～22.0 | ≥22.1 |
| 11～ | 20.3～23.5 | ≥23.6 |  | 21.1～23.2 | ≥23.3 |
| 12～ | 21.0～24.6 | ≥24.7 |  | 21.9～24.4 | ≥24.5 |
| 13～ | 21.9～25.6 | ≥25.7 |  | 22.6～25.5 | ≥25.6 |
| 14～ | 22.6～26.3 | ≥26.4 |  | 23.0～26.2 | ≥26.3 |
| 15～ | 23.1～26.8 | ≥26.9 |  | 23.4～26.8 | ≥26.9 |
| 16～ | 23.5～27.3 | ≥27.4 |  | 23.7～27.3 | ≥27.4 |
| 17～ | 23.8～27.7 | ≥27.8 |  | 23.8～27.6 | ≥27.7 |
