# Supplementary material for: Prevalence of high blood pressure subtypes and its associations with BMI in Chinese children: a national cross-sectional survey
Source: BMC Public Health. 2017 Jun 26;17:598. doi: 10.1186/s12889-017-4522-2 (PMC5485696; doi:10.1186/s12889-017-4522-2)
Supplement: Supplementary file 2 — Body Mass Index Reference Norm for Screening underweight Among Chinese children aged 6–17 years (kg/m2). (DOC 87 kb) [file 12889_2017_4522_MOESM2_ESM.doc]

| **Table S2. Body Mass Index Reference Norm for Screening underweight Among Chinese children aged 6-17 years (kg/m^2^)** | | |
| --- | --- | --- |
| Age | Boys | Girls |
| 6.0～ | 13.4 | 13.1 |
| 6.5～ | 13.8 | 13.3 |
| 7.0～ | 13.9 | 13.4 |
| 7.5～ | 13.9 | 13.5 |
| 8.0～ | 14.0 | 13.6 |
| 8.5～ | 14.0 | 13.7 |
| 9.0～ | 14.1 | 13.8 |
| 9.5～ | 14.2 | 13.9 |
| 10.0～ | 14.4 | 14.0 |
| 10.5～ | 14.6 | 14.1 |
| 11.0～ | 14.9 | 14.3 |
| 11.5～ | 15.1 | 14.5 |
| 12.0～ | 15.4 | 14.7 |
| 12.5～ | 15.6 | 14.9 |
| 13.0～ | 15.9 | 15.3 |
| 13.5～ | 16.1 | 15.6 |
| 14.0～ | 16.4 | 16.0 |
| 14.5～ | 16.7 | 16.3 |
| 15.0～ | 16.9 | 16.6 |
| 15.5～ | 17.0 | 16.8 |
| 16.0～ | 17.3 | 17.0 |
| 16.5～ | 17.5 | 17.1 |
| 17.0～ | 17.7 | 17.2 |
| 17.5～18.0 | 17.9 | 17.3 |
