# Supplementary material for: Prevalence of high blood pressure subtypes and its associations with BMI in Chinese children: a national cross-sectional survey
Source: BMC Public Health. 2017 Jun 26;17:598. doi: 10.1186/s12889-017-4522-2 (PMC5485696; doi:10.1186/s12889-017-4522-2)
Supplement: Supplementary file 3 — Showed the prevalence of HBP (%) according to age groups in Chinese 6–17 years old children, before and after adjusted for BMI and age. HBP(high blood pressure). **The original prevalence of HBP subtype is significantly different between boys and girls (≤0.001). *The original prevalence of HBP subtype is significantly different between boys and girls (≤0.05). (PPT 187 kb) [file 12889_2017_4522_MOESM3_ESM.ppt]

## Slide 1
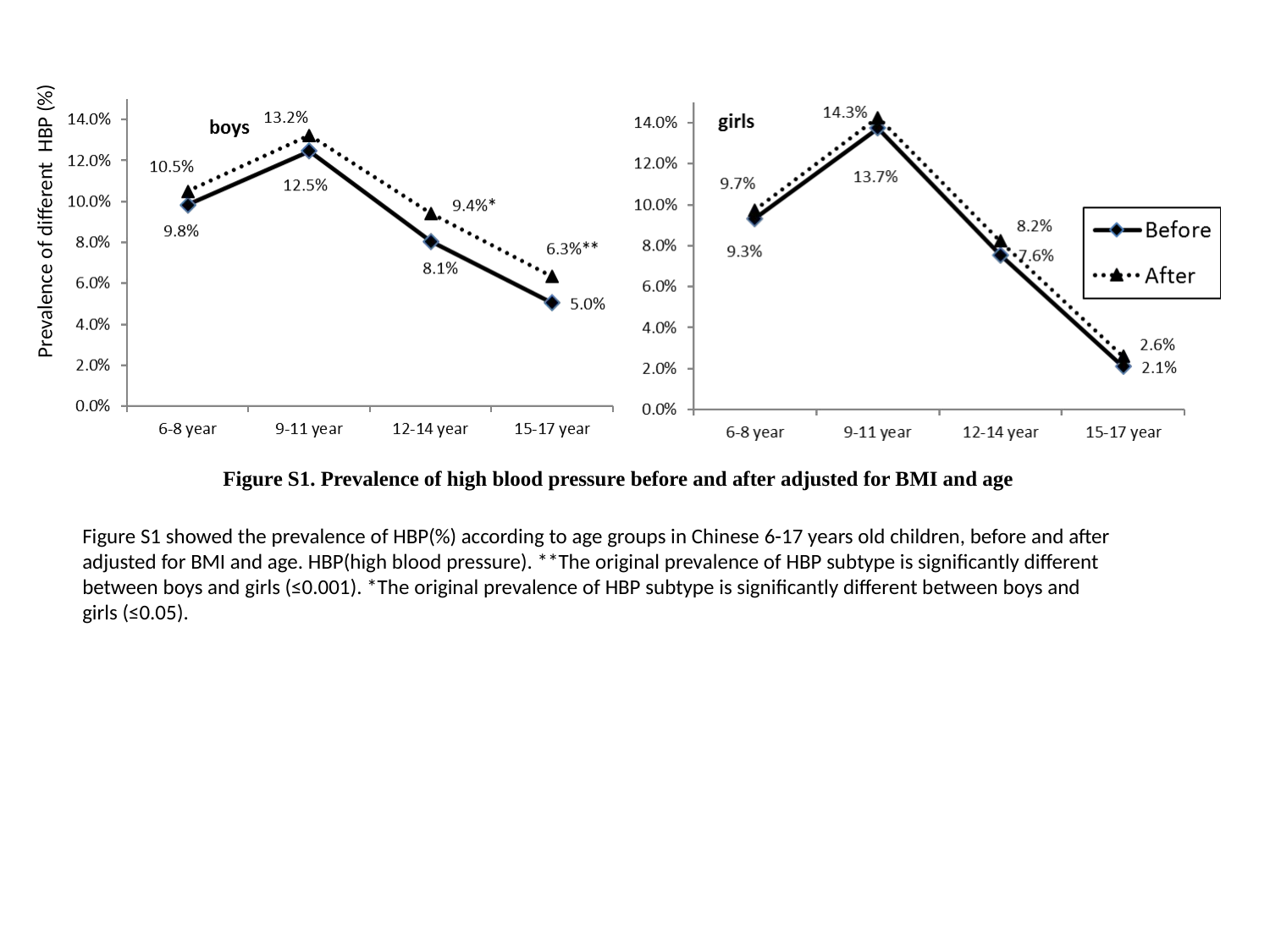

Prevalence of different HBP (%)
6.0%
Figure S1. Prevalence of high blood pressure before and after adjusted for BMI and age
Figure S1 showed the prevalence of HBP(%) according to age groups in Chinese 6-17 years old children, before and after adjusted for BMI and age. HBP(high blood pressure). **The original prevalence of HBP subtype is significantly different between boys and girls (≤0.001). *The original prevalence of HBP subtype is significantly different between boys and girls (≤0.05).
