# Supplementary material for: Prevalence of high blood pressure subtypes and its associations with BMI in Chinese children: a national cross-sectional survey
Source: BMC Public Health. 2017 Jun 26;17:598. doi: 10.1186/s12889-017-4522-2 (PMC5485696; doi:10.1186/s12889-017-4522-2)
Supplement: Supplementary file 4 — The prevalence of HBP or different HBP subtypes among 6–17 years old Chinese schoolchildren. (DOC 47 kb) [file 12889_2017_4522_MOESM4_ESM.doc]

| **Table S3. The prevalence of HBP or different HBP subtypes among 6-17 years old Chinese schoolchildren.** | | | | | | | | |
| --- | --- | --- | --- | --- | --- | --- | --- | --- |
| Age(years) | Boys | | | | Girls | | | |
|  | ISHBP | IDHBP | SDHBP | HBP | ISHBP | IDHBP | SDHBP | HBP |
| 6 | 2.0% | 3.3% | 2.4% | 7.7% | 2.3% | 3.9% | 2.1% | 8.3% |
| 7 | 3.4% | 3.2% | 3.7% | 10.3% | 2.9% | 3.0% | 3.4% | 9.3% |
| 8 | 4.9% | 4.2% | 3.5% | 12.6% | 2.8% | 4.2% | 4.3% | 11.3% |
| 9 | 5.7% | 3.7% | 3.7% | 13.2% | 3.4% | 4.4% | 5.2% | 13.0% |
| 10 | 5.7% | 3.9% | 3.8% | 13.3% | 4.3% | 6.9% | 4.7% | 15.9% |
| 11 | 5.8% | 3.7% | 3.4% | 12.9% | 3.6% | 6.6% | 3.7% | 13.9% |
| 12 | 4.7% | 2.3% | 1.8% | 8.7% | 3.3% | 3.3% | 2.1% | 8.8% |
| 13 | 5.1% | 2.6% | 1.9% | 9.6% | 3.7% | 2.9% | 1.8% | 8.4% |
| 14 | 5.6% | 2.8% | 2.2% | 10.5% | 3.3% | 1.7% | 1.5% | 6.4% |
| 15 | 3.4% | 1.4% | 1.0% | 5.8% | 1.6% | 0.7% | 0.6% | 3.0% |
| 16 | 3.0% | 2.5% | 1.2% | 6.8% | 1.0% | 0.5% | 0.5% | 2.0% |
| 17 | 1.8% | 3.2% | 1.5% | 6.5% | 1.9% | 0.9% | 0.3% | 3.2% |
| Total | 4.4% | 3.1% | 2.7% | 10.2% | 2.9% | 3.3% | 2.7% | 8.9% |
| Note: SDHBP (combined systolic/diastolic high blood pressure),IDHBP(isolated diastolic high blood pressure), ISHBP(isolated systolic high blood pressure). | | | | | | | | |
